# Current role of artificial intelligence in head and neck cancer surgery: a systematic review of literature

**DOI:** 10.37349/etat.2023.00174

**Published:** 2023-10-24

**Authors:** Antonella Loperfido, Alessandra Celebrini, Andrea Marzetti, Gianluca Bellocchi

**Affiliations:** University of Campania “L. Vanvitelli”, Italy; ^1^Otolaryngology Unit, San Camillo Forlanini Hospital, 00152 Rome, Italy; ^2^Department of Otolaryngology Head and Neck Surgery, Fabrizio Spaziani Hospital, 03100 Frosinone, Italy

**Keywords:** Artificial intelligence, machine learning, head and neck cancer, head and neck surgery, head and neck squamous cell carcinoma

## Abstract

**Aim:**

Artificial intelligence (AI) is a new field of science in which computers will provide decisions-supporting tools to help doctors make difficult clinical choices. Recent AI applications in otolaryngology include head and neck oncology, rhinology, neurotology, and laryngology. The aim of this systematic review is to describe the potential uses of AI in head and neck oncology with a special focus on the surgical field.

**Methods:**

The authors performed a systematic review, in accordance with the Preferred Reporting Items for Systematic reviews and Meta-Analyses (PRISMA) guidelines, in the main medical databases, including PubMed, Scopus, and Cochrane Library, considering all original studies published until February 2023 about the role of AI in head and neck cancer surgery. The search strategy included a combination of the following terms: “artificial intelligence” or “machine learning” and “head and neck cancer”.

**Results:**

Overall, 303 papers were identified and after duplicate removal (12 papers) and excluding papers not written in English (1 paper) and off-topic (4 papers), papers were assessed for eligibility; finally, only 12 papers were included. Three main fields of clinical interest were identified: the most widely investigated included the role of AI in surgical margins assessment (7 papers); the second most frequently evaluated topic was complications assessment (4 papers); finally, only one paper dealt with the indication of salvage laryngectomy after primary radiotherapy.

**Conclusions:**

The authors report the first systematic review in the literature concerning the role of AI in head and neck cancer surgery. An increasing influx of AI applications to clinical problems in otolaryngology is expected, so specialists should be increasingly prepared to manage the constant changes. It will always remain critical for clinicians to use their skills and knowledge to critically evaluate the additional information provided by AI and make the final decisions on each patient.

## Introduction

Artificial intelligence (AI) is a new field of science in which computers will provide decision-supporting tools to help doctors make difficult clinical choices [1]. The achievement of a large number of health data and the desire to be able to make predictions about such data has generated considerable interest in machine learning (ML). ML represents a subset of AI that enables computers to learn from data and experiences and to act without being specifically programmed [2].

Clinical applications of ML include improving cancer diagnosis and prognosis prediction by integrating clinical and genomic data; computer vision algorithms enable for example the delineation of surgical anatomy, quick detection of radiographic abnormalities, or the classification of malignant tissue in pathological specimens such as fine-needle aspirate samples or intraoperative frozen sections [3–5].

Recent ML applications in otolaryngology include head and neck oncology (classification of malignant tissue based on radiographic and histopathologic features), rhinology (standardization of imaging reporting), neurotology (codification of adult hearing loss types), and laryngology (classification of vocal disorders) [6].

In the field of head and neck oncology, AI is showing broad potential for both diagnostic and therapeutic management. Regarding diagnosis, ML methods have been extensively investigated in all imaging modalities including ultrasound (US), computed tomography (CT), magnetic resonance (MR), and nuclear medicine [7, 8].

Interestingly some authors have recently proposed a classification neural network to distinguish normal tissue from cancerous tissue of head and neck using hyperspectral imaging (HSI). HSI is a non-invasive diagnostic modality that provides information about tissue pathology by measuring the reflected, fluorescent, and transmitted light that interacts with tissue [9].

Another promising role of ML in head and neck oncology is for automated radiotherapy planning. In fact, ML applications include several phases of the entire radiotherapy process such as auto-contouring, planning, and delivery (adaptive therapy) both for external beam radiotherapy and interventional radiotherapy (brachytherapy) [10, 11].

So far, one of the topics that have not yet been adequately investigated is the use of AI in head and neck cancer from the surgical perspective. The aim of this systematic review is to describe the potential uses of AI in head and neck oncology with a special focus on the surgical field.

## Materials and methods

**Figure 1:**
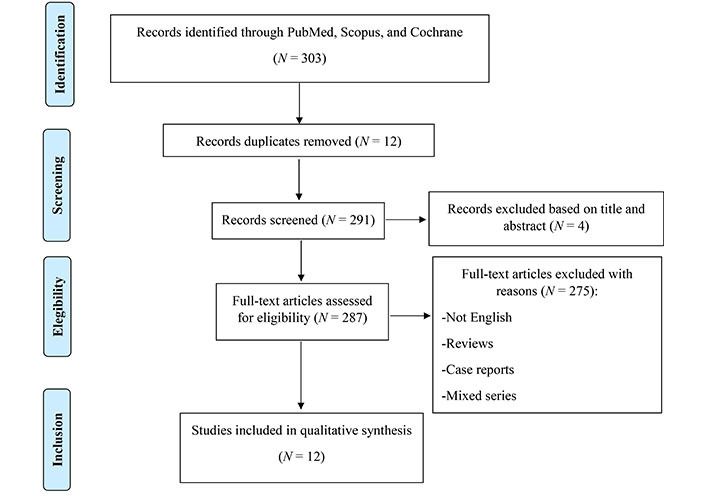
Search strategy

The author conducted this systematic review following the Preferred Reporting Items for Systematic reviews and Meta-Analyses (PRISMA) guidelines [12] as reported in Figure 1. The authors searched all papers in the three major medical databases, such as Scopus (Elsevier), PubMed [National Institutes of Health’s National Library of Medicine (NLM NIH)], and Cochrane Library (Wiley). Regarding the time period considered, we analysed all the published articles available within the databases from their inception until February 2023. In addition, the authors searched manually the main literature in head and neck conferences and eventually performed a citation chaining strategy so as not to miss any relevant articles.

The search was carried out using a combination of the following keywords: “artificial intelligence” or “machine learning” and “head and neck cancer”. The author considered as inclusion criteria for the research original articles specifically reporting on the role of AI in head and neck cancer surgery, including both prospective and retrospective studies. We excluded articles not in English, letters to the editor, conference papers, reviews, and papers off-topic. Two independent authors (AL and AC) examined titles and abstracts with the aim of finding eligible articles. The identified articles were subsequently retrieved for full-text analysis. In cases of disagreement, a panel discussion among the other authors involved with this task allowed to solve the situation. The data extracted were the authors of the paper, the year of publication, the authors’ country, the patients’ number, and the topic assessed.

## Results

The search strategy was performed according to the PRISMA guidelines. On the whole, 303 articles were found and after removing duplicate (12 papers) and excluding articles not written in English (1) and off-topic (4), articles were assessed for eligibility; finally, only 12 articles were included and summarized in Table 1.

**Table 1:** Main features of the studies included in the present systematic review

**Topic**	**Reference**	**Year**	**Country**	***N* of patients**	**Topic**
Surgical margins assessment	Costantino et al. [13]	2023	Italy	453	Prediction of positive surgical margins
	Pertzborn et al. [14]	2022	Germany	7	Intraoperative assessment of tumour margins
	Tighe et al. [15]	2021	UK	1,316	Surgical margins after curative surgery
	Halicek et al. [16]	2019	USA	102	HSI for cancer margin detection
	Halicek et al. [17]	2019	USA	12	HSI for cancer detection
	Lu et al. [18]	2017	USA	36	HSI to detect cancers
	Fei et al. [19]	2017	USA	16	HSI for tumour margin assessment
Complications assessment	Tighe et al. [20]	2022	UK	1,593	Free flap failure rates
	Mascarella et al. [21]	2022	Canada	43,701^*^	Major postoperative adverse events (MPAEs)
	Gan et al. [22]	2022	China	632	Risk factors for surgical site infection (SSI)
	Formeister et al. [23]	2020	USA	364	Complications in microvascular free tissue transfer
Salvage surgery indication	Smith et al. [24]	2020	USA	16,440	Predicting salvage laryngectomy

^*^ Number of surgical interventions. *N*: number

Three main fields of clinical interest were identified: the most widely investigated included the role of AI in surgical margins assessment (7 articles); the second most frequently evaluated topic was about complications assessment (4 papers); finally, only one paper dealt with the indication of salvage laryngectomy after primary radiotherapy.

The papers collected in this systematic review have a publication range from 2017 to 2023; with regard to the number of patients included, the authors could identify a significant variation among the studies, from less than ten to several thousand. The most widely investigated field includes the role of AI in surgical margins assessment.

Costantino et al. [13] developed and validated six ML prediction models to predict the risk of surgical positive margins in patients who underwent transoral robotic surgery (TORS). In particular, the authors found that tumour classification and tumour site are the most important predictors of positive surgical margins.

Tighe et al. [15] proposed a classification model to predict tumour margins positivity (defined as < 1 mm) using data on preoperative demographics, operations, functional status, and tumour stage. In particular, they identified three variables related to the risk of positive margins: tumour classification, extracapsular spread, and subsite of tumour. These factors are related to the biology of the disease, its growth rate, and pattern of spread in the context of local anatomy.

Other papers introduce ML HSI as a potential new approach for tumour margins assessment [14, 16–19]. In 2017, Lu et al. [18] described for the first time the utility of HIS for head and neck cancer detection highlighting that HIS, combined with ML-based quantification methods, could provide an objective, fast, and cost-effective tool to allow real-time assessment of complete resection margins.

Pertzborn et al. [14] showed that HIS on unstained fresh-frozen cancer samples in combination with an automated, deep-learning-based tumour classification model could be a potential new tool for intraoperative tumour margin assessment.

Moreover, Halicek’s research [16, 17] demonstrated that HIS combined with ML can identify head and neck squamous cell carcinoma (HNSCC) margins in surgical specimens within minutes, thus helping surgeons, reducing inappropriate surgical margins during squamous cell carcinoma (SCC) resections, and encouraging further exploration into the ability of HSI for cancer detection. Finally, Fey et al. [19] concluded that HSI technology offers great potential for cancer detection and image-guided surgery.

The second most frequently evaluated topic was complications assessment. In this regard, Formeister et al. [23] firstly described how ML can be used in head and neck microvascular reconstruction. They reported that ML manages to precisely predict the complications of head and neck free tissue transfer with a range of accuracy from 65% to 75%. Furthermore, they pointed out important elements associated with outcomes stating that we should always consider the presence of a surgical learning curve when dealing with free tissue transfer; relevant factors associated with such learning curve include the experience of the surgeon as well as the experience of the resident and of the nursing care.

Subsequently, Tighe et al. [20] addressed this issue reporting a risk adjustment algorithm to predict free flap failure rates after immediate reconstruction of head and neck defects. Mascarella et al. [21] analysed over forty thousand head and neck operations to verify which factors are most related to MPAEs. They concluded that surgical, comorbid, and frailty-related factors were most predictive of short-term MPAEs after surgical intervention. With regard to age, the authors found out that it is a poor predictor of MPAEs. Gan et al. [22] proposed the use of ML to predict the risk factors for SSI. They concluded that the three risk factors most closely associated with SSI are diabetes mellitus, the floor of the mouth as the primary tumour site and flap failure.

Finally, one paper dealt with the potential role of AI to predict which patients will require salvage total laryngectomy after primary radiotherapy with or without chemotherapy for laryngeal SCC by applying ML techniques to information from more than 16,000 patient experiences and paying particular attention to sociodemographic variability [24].

## Discussion

Head and neck surgery is considered the most ancient subspecialty in medicine and offers great opportunities for ML technology application [6]. Regarding the possible applications of AI in head and neck oncology surgery, there are three main areas of clinical interest addressed in the literature. The most widely investigated field includes the role of AI in surgical margins assessment. It is, in fact, commonly accepted that margin status is not only related to tumour biology, but also to the type of surgical approach. Radical surgery needs an adequate margin of healthy tissue around the tumour. Such a topic is of paramount importance since an adequate surgical removal of the primary HNSCC is crucial for recurrence reduction and therefore for patient outcomes including survival and quality of life [25]. A margin is defined as positive if invasive carcinoma is present at the cut tissue edge. If the tumour is within a defined distance from the cut edge, the margin is considered close. Finally, the margin is negative if the section between the tumour and the cut edge is more than that defined distance [26]. According to literature reviews, in vocal cord surgery of HNSCC, a close margin could be considered to be ≤ 1 mm, in the larynx ≤ 5 mm, in the oral cavity ≤ 4 mm, and in the oropharynx ≤ 5 mm. The choice to extend the close margin should be evaluated based on general conditions, tumour stage, and functional issues to define appropriate adjuvant therapies for each patient [27]. A common practice regarding margin status assessment is intraoperative consultation between a surgeon and a pathologist. Recently, some authors have highlighted that assessing intraoperative margins may be inadequate due to the features of the tissue sent for intraoperative analysis. In fact, extemporaneous biopsies can often be small, fragmented, unoriented, and unrepresentative of the actual margin status. Therefore, risk models should be designed and validated to define for each patient what represents a safe margin and how to judge the quality of margin revision [28].

Concerning complications assessment in HNSCC surgery, one of the potential risk factors is comorbidity, as many patients with HNSCC have concomitant diseases. Another factor to consider is the duration of anaesthesia [29]. Regarding specifical complications after reconstruction of defects with microvascular free flap transfer, the variables related to failure include comorbidities, previous oncological treatment received (chemotherapy/radiotherapy), smoking, age, intraoperative fluid administration, and overall surgery duration. More recent papers also describe additional causes of failure such as alcohol habits, prolonged ischemia, intraoperative pedicle revision, and reconstruction of the larynx. Interestingly, the high risk of failure in the laryngeal reconstruction may be associated with salivary drop and the lack of external monitoring of the skin paddle [30]. In general terms modern radiation therapy has much improved in terms of technical possibilities thus allowing for higher responses and fewer toxicities [31, 32], however, salvage total laryngectomy still represents the standard therapeutic option in the case of recurrence after radiation therapy for larynx cancer [33].

To the very best of our knowledge, this is the first systematic review in the literature concerning the role of AI in head and neck cancer surgery. However, there are some limitations in this systematic review: there are still few manuscripts about the role of AI in head and neck cancer surgery and the fields of application that can be found so far are very scanty. Consequently, the practical role of AI in head and neck cancer surgery has yet to be well-defined and further research is needed. An increasing influx of AI applications to clinical problems in otolaryngology is expected, so specialists should be increasingly prepared to manage the constant changes. It will always remain critical for clinicians to use their skills and knowledge to critically evaluate the additional information provided by AI and make the final decisions on each patient.

## Abbreviations

AI: artificial intelligence
HNSCC: head and neck squamous cell carcinoma
HSI: hyperspectral imaging
ML: machine learning
MPAEs: major postoperative adverse events
PRISMA: Preferred Reporting Items for Systematic reviews and Meta-Analysis
SSI: surgical site infection

## Declarations

### Copyright

© The Author(s) 2023.
